# Blood pressure measurement in children

**DOI:** 10.1186/1824-7288-41-S2-A19

**Published:** 2015-09-30

**Authors:** Ciro Corrado

**Affiliations:** 1 Pediatric Nephrology. “G. Di Cristina” Hospital. Palermo, Italy

## 

In pediatric age diagnosis of hypertension is defined using a statistical criterion, the limit being the 95th percentile of the distribution of the blood pressure values, according to gender, age and height [1]. Pre-hypertension is defined as blood pressure (BP) values consistently above or equal to the 90th percentile, but lower than the 95th [Table 1]. The “gold standard” method to measure BP in children is auscultatory, using an aneroid non-mercury manometer (proscribed due to their toxicity). The aneroid devices need to be calibrated every six months. At least three measurements performed on different occasions are necessary for the diagnosis of hypertension [2]. Children above 3 years of age should have their blood pressure measured every year. In all children including the youngers ones blood pressure should be measured under special circumstances that increase the risk for hypertension: intensive neonatal care, renal disease, treatment with drugs known to increase blood pressure, evidence of elevated intracranial pressure. The cuff should be of the appropriate size for the children's upper arm. Small cuffs tend to overestimate while large cuffs underestimate. The width of the cuff should be 40% of the arm circumference at a point midway between the olecranon and the acromion. The children should be calm and relaxed, seated with their right arm resting at heart level. Systolic blood pressure is defined by the first Korotkoff sound (K1) whereas diastolic blood pressure coincides with the disappearance of the pulse (K5). The use of oscillometric devices is increased in the last years. Despite their large use, only few, reported on the internet site http://www.dableducational.org were been validated in children. A diagnosis of hypertension based on an oscillometric measurement should be confirmed by an auscultatory method. Currently other two methods are used in children to measure BP values: ambulatory blood pressure monitoring (ABPM) and home blood pressure measurement. In children the use of ABPM has significant limitations due to the lack of reference values. It allows to identify “white coat hypertension” (elevated office BP values and normal ABPM values), “masked hypertension” (normal office BP values and elevated ABPM values) and subjects with or without reduced physiological day-night blood pressure variations (dipping) [3]. A new methods is represented by the self-measurement of blood pressure at home [4]. Even in this case available data from children are scanty. Correct self-measurement requires two measurements within a few minutes, performed in the morning and in the evening for 3 consecutive days.

**Table 1:** Definition and classification of hypertension in children and adolescents

Category	Systolic or diastolic blood pressure percentile
Normal	<90^th^
Pre-hypertension	≥ 90^th^and < 95^th^ ≥ 120/80 mmHg independently of the 90^th^percentile value in adolescents
Stage 1 hypertension	≥ 95^th^and < 99^th^+ 5 mmHg
Stage 2 hypertension	≥ 99^th^+ 5 mmHg
